# The Association Between the Development of Cam Morphology During Skeletal Growth in High-Impact Athletes and the Presence of Cartilage Loss and Labral Damage in Adulthood: A Prospective Cohort Study With a 12-Year Follow-up

**DOI:** 10.1177/03635465241256123

**Published:** 2024-08-05

**Authors:** Paula A.M. Claes, David F. Hanff, Adam Weir, Noortje S. Riedstra, Harrie Weinans, Denise Eygendaal, Josh Heerey, Edwin H.G. Oei, Pim van Klij, Rintje Agricola

**Affiliations:** † Department of Orthopaedics and Sports Medicine, Erasmus University Medical Center, Rotterdam, the Netherlands; ‡ Department of Radiology and Nuclear Medicine, Erasmus University Medical Center, Rotterdam, the Netherlands; § Sports Groin Pain Centre, Aspetar Orthopaedic and Sports Medicine Hospital, Doha, Qatar; ‖ Department of Orthopaedics, University Medical Center Utrecht, Utrecht, the Netherlands; ¶ La Trobe Sport and Exercise Medicine Research Centre, School of Allied Health, Human Services and Sport, La Trobe University, Melbourne, Victoria, Australia; # Department of Sports Medicine, Isala Clinics, Zwolle, the Netherlands; Investigation performed at the Erasmus University Medical Center, Rotterdam, the Netherlands

**Keywords:** hip, femoroacetabular impingement, soccer, osteoarthritis

## Abstract

**Background:**

Cam morphology develops during skeletal growth, but its influence on cartilage and the labrum in high-impact athletes later in life is unknown.

**Purpose:**

To (1) explore the association between the presence and duration of cam morphology during adolescence and the cartilage and labral status 7 to 12 years later and (2) report the prevalence of cartilage loss and labral damage in a population of young male athletes (<32 years old) who played professional soccer during skeletal growth.

**Study Design:**

Cohort study (Prognosis); Level of evidence, 2.

**Methods:**

A total of 89 healthy male academy soccer players from the Dutch soccer club Feyenoord (aged 12-19 years) were included at baseline. At baseline and 2.5- and 5-year follow-ups, standardized supine anteroposterior pelvis and frog-leg lateral radiographs of each hip were obtained. At 12-year follow-up, magnetic resonance imaging of both hips was performed. Cam morphology was defined by a validated alpha angle ≥60° on radiographs at baseline or 2.5- or 5-year follow-up when the growth plates were closed. Hips with the presence of cam morphology at baseline or at 2.5-year follow-up were classified as having a “longer duration” of cam morphology. Hips with cam morphology only present since 5-year follow-up were classified as having a “shorter duration” of cam morphology. At 12-year follow-up, cartilage loss and labral abnormalities were assessed semiquantitatively. Associations were estimated using logistic regression, adjusted for age and body mass index.

**Results:**

Overall, 35 patients (70 hips) with a mean age of 28.0 ± 2.0 years and mean body mass index of 24.1 ± 1.8 participated at 12-year follow-up. Cam morphology was present in 56 of 70 hips (80%). The prevalence of cartilage loss was 52% in hips with cam morphology and 21% in hips without cam morphology (adjusted odds ratio, 4.52 [95% CI, 1.16-17.61]; *P* = .03). A labral abnormality was present in 77% of hips with cam morphology and in 64% of hips without cam morphology (adjusted odds ratio, 1.99 [95% CI, 0.59-6.73]; *P* = .27). The duration of cam morphology did not influence these associations.

**Conclusion:**

The development of cam morphology during skeletal growth was associated with future magnetic resonance imaging findings consistent with cartilage loss in young adults but not with labral abnormalities.

Osteoarthritis (OA) is the most prevalent chronic joint disease, affecting an estimated 250 million people worldwide.^8,18^ For radiographic hip OA, Fan et al^11^ found a worldwide pooled prevalence of 8.55% (95% CI, 4.85%-13.18%). Cam morphology is one of the strongest predictors for end-stage hip OA, associated with an up to 10 times increased risk.^3,9^ The repetitive collision of cam morphology and the acetabular rim during motion can lead to cartilage and/or labral damage. In some, this process of mechanical abutment may lead to the development of symptoms (eg, femoroacetabular impingement syndrome) and hip OA.^3,13,14^

Osseous cam morphology starts developing in boys from 12 to 13 years of age and continues to increase in size during skeletal growth^2,4^ until proximal femoral growth plate closure.^45^ Its development is strongly associated with high-impact sports practice,^27^ with a dose-response relationship between physical activity during skeletal growth and cam morphology.^29,38,39,41^ Interestingly, as cam morphology is likely a mechanically driven adaptive response, there may be possibilities for the primary prevention of hip OA.

Previous studies investigating the relationship between cam morphology and hip OA have generally included participants aged >45 years from the general population.^3,9,22,33^ Few studies are available on this relationship among younger people and athletes. A total of 3 cross-sectional studies in asymptomatic young men^31^ and young soccer players^15,44^ found that cam morphology was associated with cartilage loss and labral defects. There is only 1 prospective study among high school athletes that found the presence of cam morphology at baseline to be associated with degenerative changes at 5-year follow-up.^49^ In elite-level young athletes, larger prospective studies (with a longer follow-up) investigating the link between cam morphology and early signs of hip OA are lacking. The effect of cam morphology during adolescence on the future health of the hip is uncertain. It is, for example, unknown at what age or in which proportion the onset of cartilage and/or labral damage occurs. Evaluating the early features of hip OA in young athletes may provide insights into the importance and optimal timing of preventive strategies.

The primary aim of this study was to follow a cohort of adolescent elite-level high-impact male athletes to explore the effect of the presence and duration of cam morphology on hip cartilage and labrum 7 to 12 years later. The secondary aim was to report the prevalence of early hip OA features in a population of young male athletes (<32 years old) who participated in a high-impact sport at an elite level during skeletal growth.

## Methods

### Study Design and Participants

The current study is a 12-year follow-up of an ongoing prospective cohort study that commenced in May 2010. At baseline, 89 male soccer players of the Feyenoord soccer club in Rotterdam, the Netherlands, aged between 12 and 19 years and playing in the academy (highest level for this age category), were included. Exclusion criteria for the players were any childhood hip disorder (ie, hip dysplasia, slipped capital femoral epiphysis, Perthes disease).^2,4^ One participant underwent arthroscopic cam morphology resection at 6 years after baseline.

Ethical approval for this prospective study was granted by the medical ethical committee of the Erasmus University Medical Center. Written informed consent was received from each participant.

**Figure 1.**
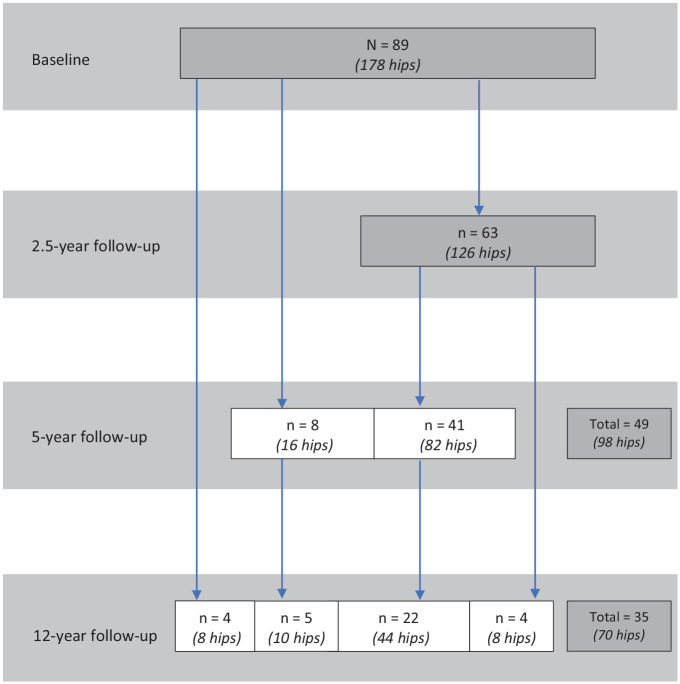
Flowchart of all analyzed participants at baseline and 2.5-, 5-, and 12-year follow-ups. Dark gray boxes represent the total number of participants at each time point.

Visits were held at baseline and 2.5-, 5-, and 12-year follow-ups. Of the 89 participants (178 hips) included at baseline, 63 participants (71%; 126 hips) were followed up at 2.5 years, 49 participants (55%; 98 hips) at 5 years, and 35 participants (39%; 70 hips) at 12 years (Figure 1).

Of the 63 participants who participated at 2.5-year follow-up, 41 continued to take part at 5-year follow-up. Of note, 4 of the 63 participants at 2.5-year follow-up were temporary dropouts; they did not take part at 5-year follow-up but rejoined at 12-year follow-up. At 12-year follow-up, participants dropped out for various reasons: 32 were unreachable, 14 rejected the invitation, 4 lived abroad, 2 accepted the invitation but did not show up, and 2 terminated during the acquisition of magnetic resonance imaging (MRI) because of claustrophobia.

Demographic data (age, height, and weight) of each participant were collected at all attended visits. At 12-year follow-up, International Hip Outcome Tool–12 (iHOT-12) scores were collected from all participants. The iHOT-12 is a questionnaire that measures health-related quality of life in young, active patients with hip disorders and consists of 4 domains: symptoms and functional limitations; sport and recreational activities; job-related concerns; and social, emotional, and lifestyle concerns. Patients self-rated the items in the domains using a visual analog scale from 0 to 100, with a score of 100 indicating having the best function and the least number of symptoms possible. The overall mean of all items equated to the final iHOT-12 score.

### Identification of Cam Morphology

At baseline and 2.5- and 5-year follow-ups, 3 radiographs^2,4,45^ were obtained from each participant per visit: a standardized supine anteroposterior view of the pelvis and a frog-leg lateral view of each hip separately.

The alpha angle was measured on all radiographs from the 3 visits. The alpha angle was automatically calculated from a manually positioned set of points that outlined the shape of the proximal femur, as described previously.^2,4^ Manual positioning of the set of points was performed by 2 authors, at baseline (R.A.) and at 2.5- and 5-year follow-up (P.K.). This method has previously shown intraclass correlation coefficients (ICCs) for intraobserver reliability that ranged from 0.85 to 0.99 and an ICC for interobserver reliability of 0.73.^5^

Cam morphology was classified as present if the alpha angle was ≥60°^46^ at any of these 3 visits. If no cam morphology was present at 1 of these visits, the proximal femoral growth plate status at the latest time point available was determined. If the growth plate was closed, it was assumed that no cam morphology would develop from that time onward,^43^ but if the growth plate was still open, the presence/absence of cam morphology was determined on MRI at 12-year follow-up using imaging planes simulating the anteroposterior pelvis and frog-leg lateral views. The physeal status was evaluated by a musculoskeletal radiologist and an orthopaedic surgeon, who were both blinded to earlier radiographic and clinical findings.

To examine the influence of the duration of cam morphology, hips with cam morphology were divided into 2 groups. Hips with cam morphology at baseline or at 2.5-year follow-up were classified as having a “longer duration” of cam morphology. Hips with cam morphology only present since 5-year follow-up were classified as having a “shorter duration” of cam morphology.

### MRI Acquisition and Scoring

#### Magnetic Resonance Imaging

At 12-year follow-up, MRI of both hips was performed on a 3.0-T scanner (SIGNA Premier; GE Healthcare). All participants were positioned supine with feet internally rotated and forefeet taped together. For each participant, a weighted pillow was placed between the ankles to ensure a reproducible position throughout the duration of scanning. A 16-channel flex coil was placed over the pelvis. Both hips were scanned separately following a standardized MRI protocol.^23^ A radial sequence was added to this protocol, resulting in the following set of sequences:

- oblique sagittal proton density–weighted fat-suppressed (3.5-mm slice thickness),
- oblique coronal proton density–weighted fat-suppressed (3.5-mm slice thickness),
- oblique axial T2-weighted fat-suppressed (3.5-mm slice thickness), and
- radial proton density–weighted fat-suppressed (2.0-mm slice thickness).

#### Scoring Hip Osteoarthritis with MRI

**Figure 2.**
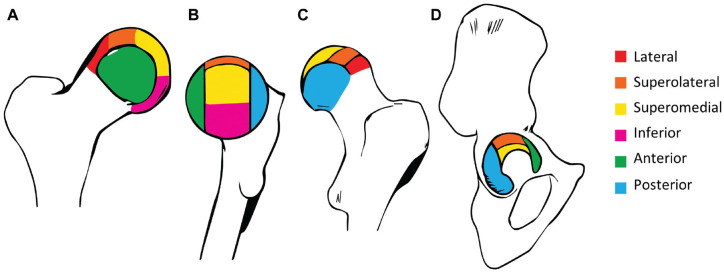
Illustration of articular subregional divisions based on the geographic zone method established by the Arthroscopy Association of North America.^21^ (A) Femoral articular cartilage subregions seen from the anterior aspect. (B) Femoral articular cartilage subregions seen from the medial aspect. (C) Femoral articular cartilage subregions seen from the posterior aspect. (D) Acetabular articular cartilage subregions seen from the lateral aspect.

The Scoring Hip Osteoarthritis with MRI (SHOMRI) tool was used to assess cartilage loss and labral abnormalities. Cartilage loss and labral abnormalities were selected for this study because they are key features of early hip OA.^25,37^ The SHOMRI is a reliable and valid semiquantitative measure developed for the evaluation of hip OA.^23^ The SHOMRI utilizes subregional divisions of the acetabulum and femoral head (Figure 2).^19^ Cartilage loss and labral abnormalities were evaluated in 10 (6 femoral and 4 acetabular) and 4 (4 acetabular) subregions, respectively.

Cartilage loss was scored on a 3-point scale: 0 for no thickness loss, 1 for partial-thickness loss, and 2 for full-thickness loss. Each subregion was scored separately to evaluate where in the hip joint cartilage loss was most common in this cohort. The sum of all 10 subregions formed the total cartilage loss score of 20. Cartilage loss was considered present if a hip had a total cartilage loss score ≥1.^16^

Labral abnormalities were scored on a 6-point scale: 0 for a healthy labrum or a normal variant (such as aplasia and hypoplasia), 1 for an abnormal signal and/or fraying of the labrum, 2 for a simple labral tear, 3 for chondrolabral separation, 4 for a complex labral tear, and 5 for maceration of the labrum. The scores of the 4 subregions were added for a total labral abnormality score of 20. A labral abnormality was defined as present if a hip had a total labral abnormality score ≥1.

All MRI scans were evaluated by an experienced musculoskeletal radiologist (D.F.H.), who was blinded to radiographic and clinical findings. Intraobserver reliability was determined using all 70 hips in this study, with scans reread at 8 months after initial scoring. Previous research on the SHOMRI has shown substantial to excellent intraobserver agreement.^12,23^

### Statistical Analysis

The difference between the baseline age of included participants and dropouts at 12-year follow-up was analyzed using the independent-samples *t* test. Differences in body mass index (BMI) and the presence of cam morphology at baseline were analyzed using the Mann-Whitney *U* test.

Intraobserver reliability for the total cartilage loss score and total labral abnormality score was determined with ICCs using a 2-way mixed model with absolute agreement. Intraobserver reliability for dichotomous cartilage loss and labral abnormality scoring was calculated with the Cohen kappa and prevalence-adjusted and bias-adjusted kappa (PABAK) and reported with positive percentage agreement.

The presence of cam morphology, cartilage loss, and labral abnormalities was reported descriptively per hip and per participant (in percentages). The prevalence of shorter and longer durations of cam morphology was reported per hip (in percentages). Total cartilage loss and labral abnormality scores were presented per hip (in percentages) and reported descriptively as the median and interquartile range. The prevalence of both cartilage loss and labral abnormalities was presented per age category for participants at 12-year follow-up.

For the evaluation of differences in the prevalence of cartilage loss and labral abnormalities between hips with and without cam morphology, logistic regression with generalized estimating equations was used. The strength of associations between the presence of cam morphology and cartilage loss or labral abnormalities was reported as the odds ratio (OR) with 95% CI and adjusted for age and BMI. All analyses were performed with SPSS Statistics (Version 28.0.1.0; IBM).

## Results

### Participants

A total of 35 participants (70 hips), with a mean age of 28.0 ± 2.0 years and mean BMI of 24.1 ± 1.8, were included at 12-year follow-up. Differences in baseline demographic data between included participants and dropouts are presented in Table 1. The mean time between baseline radiographs and MRI at 12-year follow-up was 11.9 ± 0.2 years, with a range from 11.7 to 12.4 years. At 12-year follow-up, 9 participants still played professional soccer, 20 played competitive soccer at an amateur level, and 6 no longer played soccer.

**Table 1:** Baseline Data of Patients at 12-Year Follow-up^*a*^

	Participants (n = 35)	Dropouts (n = 54)	*P*
Age, y	15.6 ± 2.2	14.8 ± 1.7	.018
BMI	20.5 ± 2.0	19.9 ± 2.4^*b*^	.236
Cam morphology, %	28.6	24.1	.638
iHOT-12 score, mean (range)	84.4 (26.3-100.0)	NA	NA

a Data are expressed as mean ± SD unless otherwise indicated. BMI, body mass index; iHOT-12, International Hip Outcome Tool–12; NA, not applicable.

b Data are missing for n = 2; thus, data for n = 52 are presented.

### Reliability

The total cartilage loss score showed excellent intraobserver reliability (ICC, 0.91 [95% CI, 0.79-0.96]; *P* < .001). Dichotomous cartilage loss scoring also showed excellent agreement (kappa, 0.90 [95% CI, 0.75-1.08]; *P* < .001) (PABAK, 1.00) with a positive agreement of 100%.

The total labral abnormality score showed good intraobserver reliability (ICC, 0.85 [95% CI, 0.65-0.94]; *P* < .001). Dichotomous labral abnormality scoring showed moderate agreement (kappa, 0.66 [95% CI, 0.37-0.95]; *P* < .001) (PABAK, 0.54) with a positive agreement of 77%.

### Prevalence of Cartilage Loss and Labral Abnormalities

**Figure 3.**
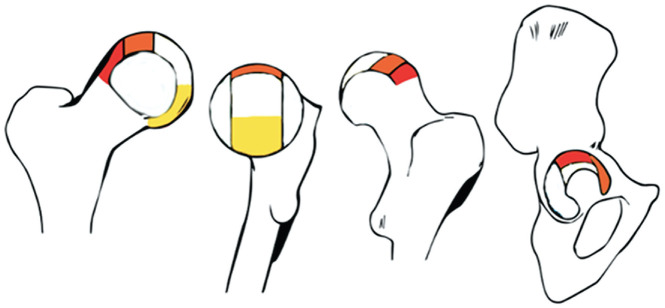
Illustration of articular subregions with the highest prevalence of cartilage loss based on the geographic zone method established by the Arthroscopy Association of North America.^19^ The red, orange, and yellow subregions are the subregions of the femur and acetabulum with the highest, second highest, and third highest prevalence of damage, respectively.

Cartilage loss was present in 33 of 70 hips (47%) and in 22 of 35 participants (63%). The median total cartilage loss score was 0.0 (IQR 0-13) (Table 2). There were 3 participants who had full-thickness cartilage loss in ≥1 subregion of 1 hip. One of these 3 had full-thickness loss in both hips. Table 3 shows the prevalence of cartilage loss, based on SHOMRI scores, by subregion of the acetabulum and femur. Cartilage loss was most prevalent in the superolateral subregion of the acetabulum and the lateral subregion of the femur. The other most affected subregions were the anterior acetabular subregion and the superolateral and inferior femoral subregions (Table 3 and Figure 3).

**Table 2:** SHOMRI Scores^*a*^

Score	Cartilage Loss (n = 70)	Labral Abnormality (n = 70)
0	37 (53)	18 (26)
1	19 (27)	18 (26)
2	5 (7)	18 (26)
3	5 (7)	2 (3)
4	1 (1)	4 (6)
5	1 (1)	1 (1)
6	00 (0)	4 (6)
7	00 (0)	00 (0)
8	00 (0)	2 (3)
9	00 (0)	2 (3)
10	00 (0)	00 (0)
11	00 (0)	00 (0)
12	1 (1)	1 (1)
13	1 (1)	00 (0)
Median (IQR)	0.0 (1)	1.0 (2)

a Data are expressed as n (%) unless otherwise indicated. SHOMRI, Scoring Hip Osteoarthritis with MRI.

**Table 3:** Cartilage Loss by Acetabular and Femoral Subregions of Hip Joint^*a*^

Subregion	No Thickness Loss (Score 0)	Partial-Thickness Loss (Score 1)	Full-Thickness Loss (Score 2)
Acetabulum (n = 70)			
Anterior	64 (91.4)	4 (5.7)	2 (2.9)
Posterior	68 (97.1)	2 (2.9)	00 (0.0)
Superomedial	68 (97.1)	2 (2.9)	00 (0.0)
Superolateral	48 (68.6)	18 (25.7)	4 (5.7)
Femur (n = 70)			
Anterior	67 (95.7)	2 (2.9)	1 (1.4)
Posterior	68 (97.1)	1 (1.4)	1 (1.4)
Superomedial	67 (95.7)	3 (4.3)	00 (0.0)
Superolateral	61 (87.1)	7 (10.0)	2 (2.9)
Lateral	56 (80.0)	14 (20.0)	00 (0.0)
Inferior	66 (94.3)	4 (5.7)	00 (0.0)

a Data are expressed as n (%).

A labral abnormality was present in 52 hips (74%) and in 31 participants (89%). A total score of 0, 1, and 2 was equally prevalent (all 26%). The median total labral abnormality score was 1.0 (IQR 0-12) (Table 2).

**Figure 4.**
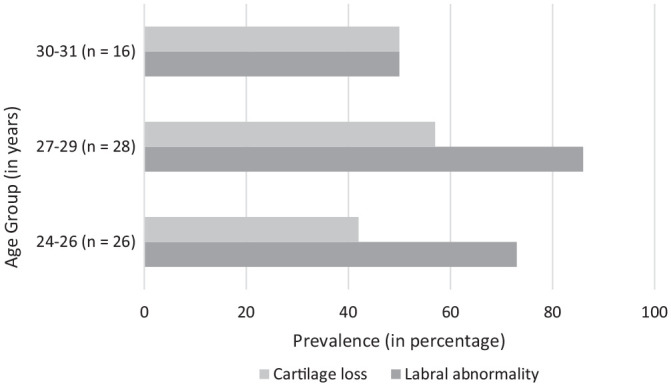
Prevalence (in percentages) of cartilage loss and labral abnormalities per age category.

Cartilage loss and labral abnormalities were present in participants of every age category, from 24 to 31 years. Figure 4 shows the prevalence of cartilage loss and labral abnormalities per age category.

### Presence of Cam Morphology and Early Hip OA Features

For 2 participants (4 hips), the alpha angle was determined on MRI at 12-year follow-up instead of on radiographs, as their latest follow-up visit with radiographs showed an open proximal femoral growth plate. Both participants had cam morphology, with one of them having it in both hips.

**Figure 5.**
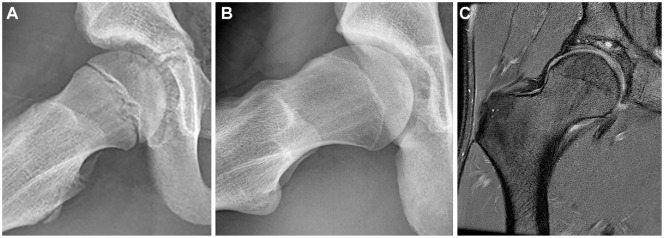
Example of a participant at (A) baseline, (B) 5-year follow-up, and (C) 12-year follow-up. (A) A frog-leg lateral radiograph of the right hip (aged 12 years) showing a spherical femoral head. (B) Presence of cam morphology at 5-year follow-up (aged 17 years). (C) Coronal proton density–weighted fat-suppressed magnetic resonance imaging of the same right hip at 12-year follow-up (aged 24 years) showing cam morphology and a local cartilage defect at the superomedial part of the acetabulum with an adjacent subchondral cyst.

**Figure 6.**
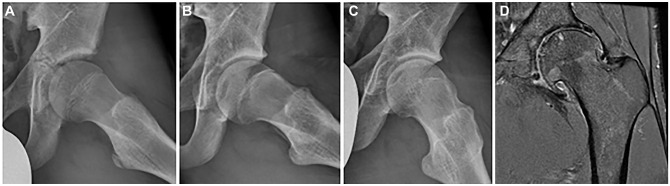
Example of a participant with 3 consecutive frog-leg lateral radiographs of the left hip at (A) baseline (aged 14 years), (B) 2.5-year follow-up (aged 16 years), and (C) 5-year follow-up (aged 19 years) showing the development of cam morphology over time. (D) Coronal proton density–weighted fat-suppressed magnetic resonance imaging of the same left hip at 12-year follow-up (aged 26 years) showing severe and diffuse acetabular and femoral cartilage loss, a subchondral cyst in the femoral head, and osteophytes.

Cam morphology was found in 56 of 70 hips (80%) and in 33 of 35 participants (94%). Cartilage loss was present in 52% of hips with cam morphology and in 21% of hips without cam morphology (adjusted OR, 4.52 [95% CI, 1.16-17.61]; *P* = .03). See Figure 5 and Figure 6 for representative examples of cam morphology development and cartilage loss at consecutive follow-up. A labral abnormality was present in 77% of hips with cam morphology and in 64% of hips without cam morphology (adjusted OR, 1.99 [95% CI, 0.59-6.73]; *P* = .27).

### Duration of Cam Morphology and Early Hip OA Features

Of the 56 hips with cam morphology, 9 hips (16%) had a shorter duration of cam morphology. A longer duration of cam morphology was present in 47 hips (84%).

Cartilage loss was present in 44% of hips with a shorter duration of cam morphology and in 53% of hips with a longer duration of cam morphology (adjusted OR, 1.36 [95% CI, 0.40-4.64]; *P* = .63). A labral abnormality was present in 67% of hips with a shorter duration of cam morphology and in 79% of hips with a longer duration of cam morphology (adjusted OR, 1.88 [95% CI, 0.36-9.78]; *P* = .45).

## Discussion

The prevalence of cartilage loss (63%) and labral abnormalities (89%) were high in this population of male former elite soccer players and already present in the youngest participants aged 24 years old. Young athletes who developed cam morphology during adolescence had 4.5 times higher odds of MRI-defined cartilage loss 7 to 12 years later than those without cam morphology. No significant association was found between hips that developed cam morphology and labral abnormalities at follow-up. Soccer players with cam morphology that was present for >10 years (longer duration) were not at a greater risk of cartilage loss and/or labral abnormalities compared with soccer players with cam morphology present between 7 and 10 years (shorter duration).

Wyles et al^49^ showed that among male and female high school athletes (with a comparable mean age at baseline to our study), those with cam morphology (alpha angle >55°) had an increased risk (relative risk, 2.5) of progressive structural changes (including cartilage and labral damage) on MRI over 5 years compared with those without cam morphology. When considering the 95% CI around this estimate (1.1-6.0), it appears comparable with our OR of 4.5. A systematic review on the relation between cam morphology and (early) hip OA could only identify prospective cohort studies among older (aged 55-65 years) participants.^43^ Nevertheless, these prospective studies, mostly from the general population, found a pooled OR of 2.52 (95% CI, 1.83-3.46) for developing hip OA when one had cam morphology, as defined by an alpha angle >60°.^9^ The strength of association in our study seems stronger; in 52% of hips with cam morphology, cartilage loss was present compared with 21% of hips without cam morphology, resulting in an adjusted OR for cartilage loss in hips with cam morphology of 4.5. This might be caused by the fact that this is an athletic population^2,4,20,30,39^ practicing high-impact impinging activities^3^ or because of the difference in imaging measures for OA. Our study used MRI, which is a more sensitive measure compared with radiography of hip OA or total hip replacement used in the previous systematic review. A recent prospective cohort study (participants aged 45-65 years) showed that cam morphology posed a 2 to 13 times increased risk for developing radiographic hip OA within 10 years, with a positive predictive value ranging from 15% for incident end-stage hip OA (Kellgren-Lawrence grade >2) to 69% for incident hip OA (Kellgren-Lawrence grade >1). This association was particularly strong for large cam morphology (alpha angle >78°) and end-stage hip OA.^42^ It is important to note that many patients with cam morphology may never develop symptoms or eventual OA. We saw that in our cohort with a high prevalence of cam morphology, most remained asymptomatic, and participants rated their hip function at a mean of 84% (mean iHOT-12 score), which is consistent with current literature.^15,17^

In line with our findings, previously published cross-sectional studies on younger and athletic populations found associations between cam morphology (presence and size) and cartilage loss. A study by Reichenbach et al^31^ in asymptomatic young men found that cam morphology was associated with reduced anterosuperior cartilage thickness. Heerey et al^15^ found an association between cam morphology size (increase in alpha angle by 1°) and cartilage loss in 237 young adult football players (adjusted OR, 1.03 [95% CI, 1.01-1.04]; *P* < .001). In the same cohort, van Buuren et al^44^ found 2 hip shape variations identified by statistical shape modeling representing cam morphology to be associated with cartilage loss. The first shape variation represented cam morphology with a less protruding greater trochanter. The second shape variation represented cam morphology with overcoverage of the acetabulum (referred to as pincer morphology) and was associated with labral abnormalities. Reichenbach et al also found associations between cam morphology and labral abnormalities, which was not found in our cohort.

The high prevalence of early OA features observed on MRI in this cohort is comparable with that in previous studies on other high-impact athletes and asymptomatic young men.^15,17,31,44,47^ Reichenbach et al^31^ found labral lesions in 72% of asymptomatic participants with and without cam morphology (mean age, 20.0 years). van Buuren et al^44^ found cartilage loss in 48% of hips and a labral abnormality in 70% of hips among a combined symptomatic and asymptomatic athletic population (median age, 26 years). These results resemble the prevalence of cartilage loss and labral abnormalities in our cohort: 47% and 74% of hips, respectively. This implies that early signs of hip OA are already present in patients with cam morphology from their early 20s.

Of the 33 hips with cartilage loss, the majority (58%) had a score of 1, meaning partial-thickness cartilage loss in only 1 of 10 subregions of the hip joint. Interestingly, most cartilage loss was found in the weightbearing superolateral subregion and anterior subregion of the acetabulum, which is the location where cam morphology impinges on the acetabulum.^6,7,21,40^ A labral abnormality was more common, with a median total labral abnormality score of 1.0 (IQR 0-12), implying 1 subregion with abnormal signaling and/or fraying of the labrum. This means that most features of cartilage loss and labral abnormalities were mild. One could even debate whether fraying or signal abnormalities should be considered a pathologic finding. It is still not certain in which proportion these mild signs of cartilage loss progress in definite hip OA. Some studies have suggested that cartilage loss on MRI expedites the development of knee OA^1,48^; this may also be the case for hip OA.

As previous studies among older people have already shown a consistent association between cam morphology and hip OA,^42^ cam morphology might be an interesting target for preventive actions. Mild cartilage defects were present in participants as young as 24 years old; therefore, preventive measures for OA should be implemented at least before the early 20s. The development of cam morphology depends on the type and frequency of loads during high-impact activities.^32,34^ Avoiding these frequent specific loading patterns while growth plates are open could contribute to preventing the development of cam morphology. Defining the line between healthy and excessive loading of the hip joint, as well as implementing these types of “deloading” activities in young athletes, can be challenging. Future research is needed on which specific loading patterns, which type of sport, and which intensity should be altered or avoided.

This study has several limitations that should be acknowledged. The high dropout rate resulted in a relatively small group attending 12-year follow-up, making it an explorative study. Still, the association between cam morphology and cartilage lesions was statistically significant, which likely indicates a strong relationship. We cannot rule out, however, that the nonsignificant association between cam morphology and labral abnormalities and the nonsignificant effect of the duration of cam morphology might be a result of a type II error. All participants in this cohort were male and came from the same youth soccer academy in the Netherlands, thereby not being representative of female athletes, other types of sports, or the general population. As cam morphology was highly prevalent in this cohort and all participants were involved in elite-level impact sports, the natural history and effect of age on hips with lower loads and normal morphology could not be assessed.

As radiographs were collected only at baseline and 2.5- and 5-year follow-ups, the duration of cam morphology could possibly be underestimated. These intervals are quite long, and therefore, it is not precisely known at what time cam morphology first started to develop. We could have missed some years of exposure in participants with cam morphology since baseline.

Finally, magnetic resonance arthrography is considered superior to unenhanced MRI for assessing cartilage and labral conditions.^26,35^ Because of ethical considerations, no intra-articular contrast was used for MRI. This could result in imaging being more sensitive in detecting labral abnormalities because any signal alteration is considered abnormal. In addition, the difference between normal and partial-thickness cartilage loss can be challenging and result in rating more positive outcomes. However, multiple studies have shown that high-resolution 3-T MRI is capable of detecting cartilage loss as well as labral abnormalities with comparable or even higher accuracy compared with magnetic resonance arthrography.^10,24,26,28,36,50^ All scans were assessed by only 1 radiologist, which may have led to underreporting or overreporting of findings.

## Conclusion

This prospective study of male elite youth soccer players found that hips that developed cam morphology during adolescence had 4.5 times increased odds of cartilage loss 7 to 12 years later. No such association was found for labral abnormalities. A longer duration of the presence of cam morphology did not seem to be associated with a higher risk of cartilage loss or labral abnormalities. A high prevalence of cartilage loss and labral abnormalities was observed in young adults aged 24 to 31 years who practiced high-intensity sports during youth, although these findings were generally mild in severity. The strong association between cam morphology and cartilage loss warrants primary or secondary preventive strategies in athletes.
